# Institutional strategies as a mechanism to rationalize the negative effects of the judicialization of access to medicine in Brazil

**DOI:** 10.1186/s12913-020-4929-9

**Published:** 2020-02-03

**Authors:** Virginia Oliveira Chagas, Mércia Pandolfo Provin, Pedro Augusto Prado Mota, Rafael Alves Guimarães, Rita Goreti Amaral

**Affiliations:** Federal University of Goiás, Goiânia, 38 Luzia Miranda St. Jataí, Goiás, 75800-000 Brazil

**Keywords:** Judicialization of health, Pharmaceutical services, Right to health, Judicial decisions, Health systems

## Abstract

**Background:**

Recently, the Executive Branch and Judiciary in Brazil increased spending due to larger numbers of lawsuits that forced the State to provide health goods and services. This phenomenon, known as health judicialization, has created challenges and required the Executive Branch and Judiciary to create institutional strategies such as technical chambers and departments to reduce the social, economic and political distortions caused by this phenomenon. This study aims to evaluate the effects of two institutional strategies deployed by a Brazilian municipality in order to cope with the economic, social and political distortions caused by the phenomenon of health judicialization regarding access to medicines.

**Methods:**

A longitudinal study was carried out in a capital in the Central-West Region of Brazil. A sample of 511 lawsuits was analyzed. The variables were placed into three groups: the sociodemographic characteristics and the plaintiffs’ disease, the characteristics of the claimed medical products and the institutional strategies. To analyze the effect of the interventions on the total cost of the medicines in the lawsuits, bivariate and multivariate linear regressions with variance were performed. For the categorical outcomes, Poisson regressions were performed with robust variance, using a significance level of 5%.

**Results:**

A reduction in the costs of medicines in the lawsuits and of the requests for medicines within the SUS formulary was verified after the deployment of the Department of Assessment of Nonstandardized Medicines (DAMNP) and the Technical Chamber of Health Assessment (CATS); an increase in processed prescriptions from the Brazilian Universal Health System was observed after the deployment of the CATS; and an increase in medicines outside the SUS formulary without a therapeutic alternative was verified after the CATS.

**Conclusion:**

The institutional strategies deployed were important tools to reduce the high costs of the medicines in the lawsuits. In addition, they represented a step forward for the State, provided a benefit to society and indicated a potential path for the health and justice systems of other countries that also face problems caused by the judicialization of health.

## Background

In Brazil, the integral right to health is an obligation of the state, written in the federal constitution, and it depends on the creation and implementation of public health policies. Brazilians have, since the 1990s, resorted to the judicial system in order to have access to medications and other health goods and services [1]. This phenomenon is called health judicialization, and drugs are the most requested good [2–4]. The causes of this phenomenon are flaws in health policies and insufficient funds for the state to meet the growing demand for health care [5].

To guarantee the effectiveness of the right to health, the Brazilian justice system established state and nonstate institutions. The former are organized into three branches: judiciary, executive, and legislative; and the latter are composed of other institutions, one of which is the public prosecutor’s office. The public prosecutor’s office is an entity that is not within these powers. It has the role of inspecting and protecting the fundamental principles and interests of society and filing lawsuits when such interests are not maintained; it is essential to justice [6].

In health care, the executive branch is responsible for managing the Brazilian public health system—the Sistema Único de Saúde (SUS)—which also guarantees access to essential medicines, guided by the National Medicines Policy [7] and by other laws and policies that define the medicines in the SUS formulary (those belonging to the official lists of medications of the SUS [8]). However, the SUS cannot meet the unlimited demands of Brazilians. When the state faces difficulties in offering health services and adequate universal well-being to all citizens, many people seek these rights through the judiciary [9]. Citizens use lawsuits to overcome lack of access to medicines, caused either by the lack of financial resources to acquire them, by their unavailability in the public health service, or by the high cost of treatment. Medicines that treat genetic diseases and cancer that are outside the pharmaceutical care policy represent an important part of the problem [5, 10].

In the Brazilian democratic context, on the one hand, health judicialization can express legitimate claims and ways of acting by citizens. On the other hand, it bolsters an imbalance in rights by giving the privilege of obtaining these rights to groups of people who have knowledge about lawsuits and the financial resources to appeal to the justice system [11].

Some studies show that those who use the judiciary to request a medicine usually come from better socioeconomic conditions, because they can pay for the procedural expenses generated by the judicialization. Thus, this phenomenon privileges certain social segments, favoring those who have greater access to the justice system, and, regarding access to health, aggravates the inequities of a system already marked by inequalities [12].

The search for a guarantee of one’s right to health services and goods using the courts is not isolated to Brazil; it occurs in other countries, such as Colombia, Costa Rica, and Chile [13–15]. What stands out in the Brazilian case is the high rate of the lawsuits deferred by judges, which has sparked discussions between professionals and managers of the health sector, as health judicialization not only can provide a useful mechanism for society to force the state to implement public policies but also can cause negative economic, social, and political distortions [4].

In economic terms, judicialization creates costly, unscheduled expenses for the executive branch and judiciary. For the executive, the spending in the federal sphere on such judicial demands has increased 1000% (in the requested items) between 2008 and 2015, increasing from approximately US$32,928,388.75 to US$351,662,404.09, which has displaced the budget for other sectors of the Ministry of Health, such as the supply of drugs to primary care and the treatment of patients with STIs and AIDS; in addition, the Ministry of Health budgets showed a limited variation in this time period, which made planning and managing the public budget difficult [16]. This is a distortion, wherein some are benefiting while others are losing; only those who win in this process benefit while others may lose and/or have no recourse.

For the judiciary, it was observed that spending on lawsuits increased to approximately US$11,319,112,008 (the administrative cost of appeals), which corresponded to 1.2% of the gross national product and was the equivalent of handling an overall procedural burden of approximately 86.6 million cases [17]. In 2011, there were 240,980 lawsuits in the health area, and they were primarily requests for medications [18].

With regard to social distortions, studies have revealed that lawsuits can deepen the inequity of access to care in a health system already characterized by socioeconomic inequality. Individuals with better socioeconomic conditions, who are not participants of the SUS and are financially able to afford lawsuits, are therefore favored. By favoring a few individuals, lawsuits interfere negatively with the principles of the SUS (universality and equity), because these demands shift resources that should serve the majority (especially those who need it the most) in order to serve a few with smaller needs [3, 5, 19].

As for political distortions, lawsuits can interfere and cause distortions in the national medicines policy and force the government to provide medicines outside the SUS formulary, with little evidence of their efficacy and safety and which have higher costs.

This may occur due to members of the judiciary and the public prosecutor’s office lacking the technical knowledge to interpret these policies. In lawsuits, there are no trials with competing experts, and the judge decides based solely on the information provided in the plaintiff’s request. In this case, the government is not given an opportunity to answer the plaintiff’s demand. The government has to comply with the judicial decision within the deadline stipulated by the judge. Such distortions can compromise the rational use of medicines [20].

Therefore, health judicialization has created tensions and motivated discussions about its legitimacy and the technical and/or legal and institutional competence of the judicial system to decide about the content and the implementation of the state provision as implemented by the executive branch. In addition, it has caused an increasing number of individual demands for medications that increase public spending [2, 3].

To rationalize these distortions, Brazil and other Latin American and Caribbean countries, such as Chile, Costa Rica, Mexico, Peru, Uruguay, and Argentina, have proposed strategies, such as the creation of spaces for discussion between the actors in the health and judicial systems to help in the decision-making process, according to the needs of the population. For this, technical and scientific consultation committees were created to aid operatives of the law before filing a health-related lawsuit. Such strategies have helped in rationalizing finite health resources, creating interinstitutional dialogue between actors in the executive branch and the judiciary, and reducing new judicial demands, but initially this caused an increase in the bureaucratic system [21–23].

In Brazil, the creation of the Department of Assessment of Nonstandardized Medicines (Departamento de Avaliação de Medicamentos Não Padronizados, or DAMNP) and the Technical Chamber of Health Assessment (Câmara de Avaliação Técnica em Saúde, or CATS) were strategies deployed by one Brazilian state. The DAMNP was a department created by the executive branch in 2006 to exclusively analyze the technical and scientific rationality of medicines solicited by the users of the public health system by using an administrative case that has a prescription and a medical report. The technical analysis was performed by pharmacists who evaluated the demands according to the norms of policies and national legislation. When the solicited medicine was outside the SUS formulary, the pharmacists suggested its replacement by one belonging to the official lists of the SUS. This strategy was responsible for establishing access protocols to medications by the executive branch and, when necessary, for the inclusion or exclusion of medicines on the official lists of the SUS [24].

The CATS was created in 2009 by the public prosecutor’s office of the Brazilian state in order to offer technical support to public prosecutors regarding medicines solicited by the users of the public and private health systems [25]. The opinions are from medical experts and pharmacists who analyze the medical prescriptions and report based on medical and scientific criteria and on pharmaceutical policies. Beyond that, these professionals, when possible, can train providers to prescribe therapeutic alternatives made available by the SUS. This has contributed to perfecting access protocols for medicines by the executive branch, suggesting medicines to be incorporated into the policies, cutting down on legal costs, and bringing evidence and expertise to the issue, rather than just having lawyers sort out the matter [25].

The purview of the CATS was reaffirmed in 2010 when a technical cooperation agreement was signed between the executive branch and the public prosecutor’s office. This agreement decreed that all demands for medicines not included in the official SUS lists would be dealt with by filing an administrative case in the executive branch. Institutionalizing these administrative cases was viewed as a way to reduce the number of lawsuits [26].

It was noted that these strategies were adopted by jurists and health managers in many Brazilian states and conveyed respect for constitutional principles, such as the right to health as an aspect of human dignity and access to justice [27–29]. However, little is known about the effects of these strategies as they pertain to negative repercussions caused by the judicialization of the health system. This gap in knowledge motivates the following question: Do institutional strategies reduce the social, economic, and political distortions that the judicialization of access to medicine brings to public administration?

In this context, the objective of this study was to evaluate the effects of two institutional strategies adopted by a Brazilian municipality in order to confront economic, social, and political distortions that involved the judicialization of access to medicine.

## Methods

### Data source and study setting

This was a longitudinal study whose objective was to assess the lawsuits that requested medicine from the executive branch before and after the deployment of two institutional strategies in a state capital in the Central-West Region of Brazil. These were deployed in order to contend with the distortions caused by the judicialization of access to medicine.

To analyze these lawsuits, the period from 2003 to 2015 was chosen. The data were collected in July of 2016 in the Pharmacy of Health-Related Products and Special Medications, a unit responsible for archiving and attending to such demands. Lawsuits that solicited at least one medicine were included in the study.

A total of 3335 lawsuits requesting health goods or services from January 2003 to December 2015 were identified; of these, 2557 solicited at least one medication.

**Fig. 1:**
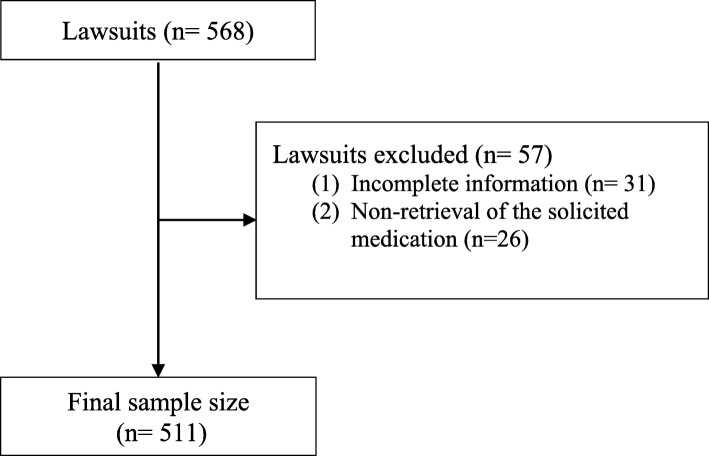
Flow chart of the sample size

The random simple type of calculation for the sample was used that was stratified by the year of the lawsuit, using a statistical power of 80 (β = 20%), a confidence interval of 95% (α = 0.05), and an accuracy level of 5%. A total of 568 lawsuits were selected, of which 31 were excluded due to incomplete information and 26 were excluded for nonretrieval of the solicited medication, yielding a total sample size of 511 lawsuits during the analyzed time period. The lawsuits that occurred during the year of the deployment of the policy (a transitional period) were removed from the analysis. Were pulled out 50 lawsuits in the CATS implementation year, and 97 lawsuits in the DAMNP implementation year (Fig. 1).

### Variables

The lawsuit data were collected using a form standardized by the researchers after reviewing variables that were investigated in a prior study done in Brazil [30], which included the following: (i) sociodemographic characteristics and the disease(s) of the plaintiffs; (ii) characteristics of the medications solicited in the lawsuits; and (iii) the institutional strategies.

(i) Sociodemographic characteristics and the disease(s) of the plaintiffs

The sociodemographic characteristics and the disease of the plaintiffs included age (years), sex (male/female), the prescription’s origin (were prescribed by a SUS clinician or private system clinician), and income (the mean monthly income of the head of the household, expressed in US dollars), estimated according to the 63 Territorial Planning Unit (Unidade Territorial de Planejamento, or UTP) of the capital and acquired from the 2010 census data [31]; disease(s) were classified according to the *International Statistical Classification of Diseases and Related Health Problems* (ICD) [32] and reported in the medical report included within the lawsuit.

(ii) Characteristics of the medications solicited in the lawsuits

The characteristics of the requested medicines in the lawsuits were as follows: the amount of medication(s) requested; the classification of the medications according to the *Anatomical Therapeutic Chemical Classification* [ATC] [33]; and the total cost of the medicines in the lawsuit (relative only to the sum of the costs of the medicines requested based on the price of the Bank of Health Prices of the Ministry of Health) [34]. Medications were also classified according to their position with regard to the official lists (i.e., if they are on the medicines list and are provided free of charge). The categories were as follows: (1) in the SUS formulary (belonging to the official lists of medications of the SUS); (2) outside the SUS formulary (per the official SUS lists of medications) but with a therapeutic alternative available from the SUS through the third level of the ATC classification (yes or no) [32]; or (3) outside the SUS formulary and without a therapeutic alternative available from the SUS (yes or no) [8].

(iii) Institutional strategies

The group of institutional strategies considered by the Department of Assessment of Nonstandardized Medicines (Departamento de Avaliação de Medicamentos Não Padronizados, or DAMNP) and the Technical Chamber of Health Assessment (Câmara de Avaliação Técnica em Saúde, or CATS).

**Fig. 2:**
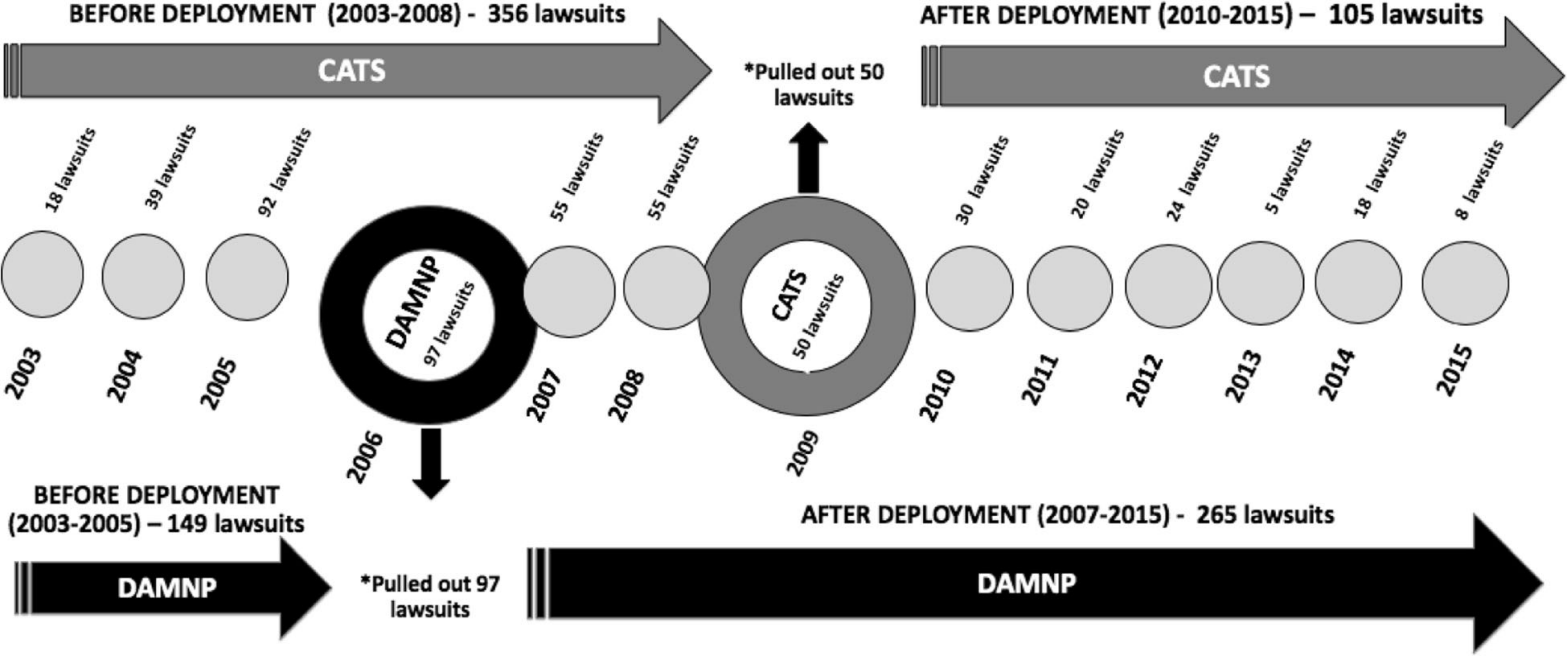
Scheme of data collection of lawsuits in the period before and after deployment of the Technical Chamber of Health Assessment (CATS) and the period before and after deployment of Department of Assessment of Non-Standardized Medicines (DAMNP). *pulled out 50 lawsuits in the CATS implementation year, and 97 lawsuits in the DAMNP implementation year

The variables related to the interventions were determined according to previously published studies [35, 36]. Two dummy variables were created in a dichotomized fashion: a preintervention period (0) and a post-intervention period (1). In this manner, the lawsuits were coded with “0” if they were filed before the deployment of each intervention and with “1” if they were filed after the deployment of the interventions. The lawsuits that occurred in the year of the intervention deployment (the transitional period) were removed from the analysis in order to avoid potential bias (Fig. 2). The coding was done by a specialist researcher. The coding of the variables analyzed in the study is shown in Table 1.

**Table 1:** Codding of the variables in the study

Variables	Operational definition	Categories or unity
Characteristics of the lawsuits		
Sex	Plaintiff’s sex	0 - Male 1 - Female
Age	Plaintiff’s age	Age in years
Income	Plaintiff’s income	Dollars
Cost of medicines	Total cost of medicines in the lawsuit	Dollars
Quantity of medicines	Total amount of medicines in the lawsuit	Number (n)
Prescription’s origin	Type of healthcare establishment in which the requested medicine was prescribed	0 – Prescribed by a SUS clinician 1 – Prescribed by a private system clinician
Diseases Plaintiff’s disease according to ICD^a^ [30]		0 - Certain infectious and parasitic diseases 1- Neoplasms
		2 - Diseases of the blood and blood-forming organs and certain disorders involving the immune mechanism
		3 - Endocrine, nutritional and metabolic diseases
		4 - Mental and behavioural disorders
		5 - Diseases of the nervous system
		6 - Diseases of the eye and adnexa
		7 -Diseases of the circulatory system
		8 - Diseases of the respiratory system 9 - Diseases of the digestive system 10 -Diseases of the musculoskeletal system and connective tissue
		11 -Diseases of the genitourinary system
Characteristics of the medicines		
ATC^b^ Classification Classification of the medicines according to the World Health Organization’s classification [31]		1. Alimentary tract and metabolism 2. Blood and blood forming organs 3. Dermatological 4. Cardiovascular system
		5. Genital and urinary system and sex hormones
		6. Systemic hormonal preparations 7. Anti-infective for systemic use
		8. Antineoplastic and immunomodulation agents
		9. Muscular-skeletal system 10. Nervous system 11. Anti-parasitic products, insecticides and repellents
		12. Sensory organs 13. Various
Classification of the medicines	Classification of medicines according to official lists of SUS^c^ [8]	
Within the SUS formulary (belonging to the official lists of medications of SUS)		0 - No 1 – Yes
Outside the SUS formulary (per the official SUS lists of medications) but with a therapeutic alternative available from SUS		0 – No 1 - Yes
Outside the SUS formulary and without a therapeutic alternative available from SUS		0 – No 1 - Yes
Interventions		
DAMNP		0 – Period before deployment (2003–2005) 1 – Period after deployment (2007–2015)
CATS		0 – Period before deployment (2003–2008) 1 – Period after deployment (2010–2015)

^a^Diseases classified according to the chapter of International Statistical Classification of Diseases and Related Health Problems (ICD); ^b^Anatomical Therapeutic Chemical; ^c^ Sistema Único de Saúde

### Statistical analysis

The data were analyzed using SATA software, version 14.0. The Shapiro-Wilk (SW) test was used to verify the normality of the quantitative variables [37]. An initial descriptive analysis of the lawsuits included in the sample was performed, and the qualitative variables were represented as absolute and relative frequencies and the quantitative variables as the mean, standard deviation (SD), median, and interquartile range (IQR) [38].

To verify the effect of the three institutional strategies on the economic and political characteristics of the lawsuits, the following were considered dependent variables: (i) the total cost of medications in the lawsuit; (ii) lawsuits with medicines in the SUS formulary; (iii) lawsuits with medicines outside the SUS formulary but with a therapeutic alternative available from the SUS; and (iv) lawsuits with medicines outside the SUS formulary and without a therapeutic alternative from the SUS. For the statistical analysis, the deployment of the strategy was entered as a variable. This variable was further categorized by period of occurrence as a pre-deployment or a post-deployment strategy. To prevent potential bias, the lawsuits that occurred during the year of the deployment of the policy (a transitional period) were removed from the analysis.

To analyze the effects of the interventions and the determinants of the total cost of the medications, bivariate and multiple linear regressions with robust variance were performed [39, 40]. The following were considered determinants in the cost of the process: the prescription’s origin, the quantity of medicines, and the disease. Variables with *p* <  0.20 in the bivariate analysis were included in the multiple regression model. The characteristics of the plaintiffs (age, sex, and income) were also included in the adjustment of the regression model. Due to the asymmetrical nature of the date related to the costs, bootstrapped standard errors (based on 1000 replications) were calculated to obtain 95% confidence intervals in order to adjust the regression coefficient [41, 42] beyond the logarithmic transformation of the data [38]. The models were analyzed for multicollinearity using the variance inflation factor (VIF) test [43], for the distribution of residues using the SW test [37], and for homoscedasticity using the White test [44].

To verify the effect of interventions on categorical outcomes, Poisson regressions with robust variance were performed [45, 46]. The models were adjusted by the following variables: the prescription’s origin and interventions. In all analyses, values of *p* <  0.05 were considered statistically significant.

## Results

In the 511 processes analyzed, 1501 medicines were requested. The mean age of the plaintiffs was 42.8 years (SD ± 24.7), 57.1% were males, the median age was 43.0 years (20.0–64.0), the mean income was US$1409.6 (SD ± 1101.8) (above poverty) and the median was US$1036.9 (680.5–1506.5), the mean cost of medications of the lawsuits was US$1483.3 (SD + 4345.7) and the median was US$406.2 (143.9–1198.6), and the mean quantity of medications requested was 2.9 per lawsuit (SD ± 2.4) and the median was 2.0 (1.0–4.0) (Table 2).

**Table 2:** Sociodemographic characteristics and diseases of plaintiffs requesting medicines from January 2003 to December 2015

Variables	*n* = 511
Sex n (%)	
Male	292 (57.1)
Female	219 (42.9)
Age (years)	
Mean (SD)^a^	42.8 (24.7)
Median (IQR)^b^	43.0 (20.0–64.0)
Income (US$)	
Mean (SD)^a^	1409.6 (1101.8)
Median (IQR)^b^	1036.9 (680.5–1506.5)
Total costs of medications in the lawsuit (US$)	
Mean (SD)^a^	1.483.3 (4.345.7)
Median (IQR)^b^	406.2 (143.9–1198.6)
Quantity of medications n (%)	
Mean (SD)^a^	2.9 (2.4)
Median (IQR)^b^	2.0 (1.0–4.0)
Prescription’s origin n (%)	
Prescribed by a SUS clinician	77 (28.2)
Prescribed by a private system clinician	196 (71.8)
Diseases^c^ n (%)	
Certain infectious and parasitic diseases	5 (1.0)
Neoplasms	7 (1.4)
Diseases of the blood and blood-forming organs and certain disorders involving the immune mechanism	4 (0.8)
Endocrine, nutritional and metabolic diseases	71 (13.9)
Mental and behavioural disorders	77 (15.1)
Diseases of the nervous system	84 (16.4)
Diseases of the eye and adnexa	17 (3.3)
Diseases of the circulatory system	109 (21.3)
Diseases of the respiratory system	17 (3.3)
Diseases of the digestive system	27 (5.3)
Diseases of the musculoskeletal system and connective tissue	28 (5.5)
Diseases of the genitourinary system	139 (27.2)

^a^Standard deviation; ^b^Interquartile range; ^c^Diseases were classified according to the chapters of International Statistical Classification of Diseases and Related Health Problems (ICD)

Regarding the origins of the prescriptions, a greater proportion of prescriptions in administrative cases were prescribed by an SUS clinician (71.8%). The most frequent diseases were of the genitourinary system (27.2%), the circulatory system (21.3%), and the nervous system (16.4%) (Table 2).

In relation to the classification of the medicines requested in the lawsuits, a majority of medicines outside the SUS formulary was observed (54.6%) as follows: 27.7% for the digestive tract and metabolism, 27.6% for the cardiovascular system, and 20.9% for the nervous system (Table 3).

**Table 3:** Characteristics of medicines requested in lawsuits from January 2003 to December 2015 (*n* = 1.501)

Variables	n (%)
ATC Classification^a^	
Alimentary tract and metabolism	416 (27.7)
Blood and blood forming organs	75 (5.0)
Cardiovascular system	414 (27.6)
Dermatological	20 (1.3)
Genital and urinary system and sex hormones	42 (2.8)
Systemic hormonal preparations	15 (1.0)
Anti-infective for systemic use	23 (1.5)
Antineoplastic and immunomodulation agents	13 (0.9)
Muscular-skeletal system	63 (4.2)
Nervous system	314 (20.9)
Anti-parasitic products, insecticides and repellents	11 (0.7)
Respiratory system	42 (2.8)
Sensory organs	47 (3.1)
Various	5 (0.4)
Classification of medicines	
Within the SUS formulary	685 (45.7)
Outside the SUS formulary with a therapeutic alternative	430 (28.6)
Outside the SUS formulary without a therapeutic alternative	386 (25.7)

^a^Anatomical Therapeutic Chemical

Tables 4 and 5 show the number of medicines requested in lawsuits by Anatomical Therapeutic Chemical classification by year (2003–2015).

**Table 4:** Number of medicines requested in lawsuits by Anatomical Therapeutic Chemical Classification by year (2003–2009)

Variables	2003 n (%)	2004 n (%)	2005 n (%)	2006 n (%)	2007 n (%)	2008 n (%)	2009 n (%)	Total n
ATC Classification								
Alimentary tract and metabolism	6 (12.8)	16 (11.7)	80 (22.0)	110 (39.0)	70 (48.3)	36 (26.9)	15 (10.7)	333
Blood and blood forming organs	2 (4.3)	3 (2.2)	25 (6.9)	8 (2.8)	9 (6.2)	7 (5.2)	10 (7.1)	64
Cardiovascular system	11 (23.4)	41 (29.9)	146 (37.5)	62 (22.0)	32 (22.1)	42 (31.3)	42 (30.0)	376
Dermatological	1 (2.1)	–	3 (0.8)	3 (1.1)	–	–	9 (6.4)	16
Genital and urinary system and sex hormones	1 (2.1)	9 (6.6)	13 (3.6)	6 (2.1)	–	2 (1.5)	6 (4.3)	37
Systemic hormonal preparations	2 (4.3)	3 (2.2)	5 (1.4)	2 (0.7)	–	1 (0.7)	1 (0.7)	14
Anti-infective for systemic use	1 (2.1)	4 (2.9)	6 (1.7)	4 (1.4)	–	3 (2.2)	4 (2.9)	22
Antineoplastic and immunomodulation agents	–	–	2 (0.6)	5 (1.8)	2 (1.4)	–	1 (0.7)	10
Muscular-skeletal system	4 (8.5)	7 (5.1)	8 (2.2)	7 (2.5)	–	6 (4.5)	8 (5.7)	40
Nervous system	16 (34.0)	37 (27.0)	53 (14.6)	59 (20.9)	30 (20.7)	27 (20.1)	30 (21.4)	252
Anti-parasitic products, insecticides and repellents	–	1 (0.7)	5 (1.4)	5 (1.8)	–	–	–	11
Respiratory system	1 (2.1)	10 (7.3)	11 (3.0)	5 (1.8)	–	4 (3.0)	8 (5.7)	39
Sensory organs	2 (4.3)	6 (4.4)	16 (4.4)	6 (2.1)	2 (1.4)	6 (4.5)	4 (2.9)	42
Various	–	–	–	–	–	–	2 (1.4)	2
Total	47 (100.0)	137 (100.0)	373 (100.0)	282 (100.0)	145 (100.0)	134 (100.0)	140 (100.0)	1258

**Table 5:** Number of medicines requested in lawsuits by Anatomical Therapeutic Chemical Classification by year (2010–2015)

Variables	2010 n (%)	2011 n (%)	2012 n (%)	2013 n (%)	2014 n (%)	2015 n (%)	Total n
ATC Classification							
Alimentary tract and metabolism	36 (52.9)	25 (43.9)	17 (29.3)	2 (28.6)	2 (5.3)	1 (4.0)	83
Blood and blood forming organs	2 (2.9)	3 (5.3)	2 (3.4)	–	2 (5.3)	2 (8.0)	11
Cardiovascular system	8 (11.8)	17 (29.8)	11 (19.0)	–	7 (18.4)	5 (20.0)	48
Dermatological	1 (1.5)	–	–	–	3 (7.9)	–	4
Genital and urinary system and sex hormones	1 (1.5)	–	2 (3.4)	–	2 (5.3)	–	5
Systemic hormonal preparations	–	–	–	–	1 (2.6)	–	1
Anti-infective for systemic use	–	1 (1.8)	–	–	–	–	1
Antineoplastic and immunomodulation agents	–	–	1 (1.7)	1 (14.3)	1 (2.6)	–	3
Muscular-skeletal system	3 (4.4)	4 (7.0)	8 (13.8)	–	8 (21.1)	–	23
Nervous system	11 (16.2)	5 (8.8)	14 (24.1)	4 (57.1)	12 (31.6)	16 (64.0)	62
Anti-parasitic products, insecticides and repellents	–	–	–	–	–	–	
Respiratory system	1 (1.5)	2 (3.5)	–	–	–	–	3
Sensory organs	4 (5.9)	–	1 (1.7)	–	–	–	5
Various	1 (1.5)	–	2 (3.4)	–	–	1 (4.0)	4
Total	68 (100.0)	57 (100.0)	58 (100.0)	7 (100.0)	38 (100.0)	25 (100.0)	253

The costs of medicines requested were less after the deployment of the DAMNP (*p* <  0.005) and the CATS (*p* <  0.001); there was a reduction in the frequency of requests for medicines in the SUS formulary after the deployment of the DAMNP (*p* = 0.039) and the CATS (*p* = 0.020); there was an increase in requests for medicines outside the SUS formulary with a therapeutic alternative after the deployment of the DAMNP (*p* = 0.028) and the CATS (*p* <  0.001); and there was an increase in lawsuits for prescriptions by an SUS clinician after the deployment of the CATS (*p* <  0.002) (Table 6).

**Table 6:** Plaintiffs’ incomes, cost of medicines of the lawsuits, origin of the prescription and classification of medicines in the lawsuits in periods before and after the deployment of the Department of Assessment of Non-Standardized Medicines (DAMNP) and Technical Chamber of Health Assessment (CATS)

Variables	DAMNP (2006)			CATS (2009)		
	Before deployment (2003–2005)	After deployment (2007–2015)	*p*	Before deployment (2003–2008)	After deployment (2010–2015)	*p*
Income of Plaintiffs						
Mean (SD)^1^	1231.4 (858.7)	1458.2 (1182.8)	0.009^3^	1393.4 (999.8)	1458.2 (1215.2)	0.575^3^
Median (IQR)^2^	1004.5 (680.5–1263.8)	1137.8 (680.5–1263.8)		1004.5 (680.5–1263.8)	972.1 (680.5–1263.8)	
Cost of medicines in the lawsuit						
Mean (SD)^1^	1.661.1 (4.561.7)	779.2 (1.589.6)	0.005^3^	1.884.1 (5.054.8)	586.7 (1.640.3)	< 0.001^3^
Median (IQR)^2^	659.7 (188.6–1622.1)	285.6 (101.0–195.8)		541.1 (191.6–1759.4)	250.3 (94.5–616.3)	
Prescription’s origin, n (%)						
Prescribed by a SUS clinician	20 (25.6)	38 (31.4)	0.382^4^	46 (22.4)	24 (43.6)	0.002^4^
Prescribed by a private system clinician	58 (74.4)	83 (68.6)		159 (77.6)	31 (56.4)	
Classification of medicines, n (%)						
Within the SUS formulary	261 (47.7)	281 (41.8)	0.039^4^	548 (49.5)	104 (41.1)	0.020^4^
Outside the SUS formulary with a therapeutic alternative	145 (26.5)	217 (32.3)	0.028^4^	289 (26.1)	94 (37.2)	< 0.001^4^
Outside the SUS formulary without a therapeutic alternative	141 (25.8)	174 (25.9)	0.963^4^	271 (24.4)	554 (21.7)	0.360^4^

^1^Standard deviation; ^2^Interquartile range; ^3^*Mann-Whitney Test;* ^*4*^Pearson’s chi-squared test

Table 7 shows the bivariate and multivariate regression of the effects of the strategies and the other determinants of the total cost of the medicines in the lawsuits. The final model explained 14.0% of the variability of the costs of the medications in the lawsuits (R2:14.0). A reduction in the costs was noted after the deployment of the DAMNP (β:-0.20; *p* <  0.001) and of the CATS (β:-0.25; *p* <  0.001) (Table 7). The income of the plaintiff and the quantity of the medications were positively and independently associated with the cost.

**Table 7:** Determinants and effects of institutional strategies on the costs of medications requested in lawsuits from January 2003 to December 2015

Variables	Bivariate regression		Multivariable regression^3^	
	β^1^ (IC 95%)^2^	*p*	β^1^ (IC 95%)^2^	*p*
Plaintiffs’ characteristics				
Age (years)	0.05 (−0.10; 0.21)	0.517		
Sex				
Male	1.00			
Female	−0.09 (− 0.38; 0.18)	0.502		
Income (US$)	0.45 (0.23; 0.66)	< 0.001	0.41 (0.22–0.61)	< 0.001
Characteristics of the lawsuits				
Quantity of medicines	0.13 (0.07; 0.19)	< 0.001	0.17 (0.12–0.23)	< 0.001
Origin of the prescriptions				
Prescribed by a SUS clinician	1.00			
Prescribed by a private system clinician	0.84 (0.42; 1.27)	< 0.001		
Diseases				
Certain infectious and parasitic diseases	−0.84 (−2.29; 0.60)	0.252		
Neoplasms	1.40 (0.18; 2.62)	0.024		
Diseases of the blood and blood-forming organs and certain disorders involving the immune mechanism	−1.84 (−3.45; −0.23)	0.025		
Endocrine, nutritional and metabolic diseases	−0.25 (− 0.80; 0.01)	0.057		
Mental and behavioural disorders	0.07 (−0.33; 0.46)	0.740		
Diseases of the nervous system	−0.26 (− 0.64; − 0.12)	0.178		
Diseases of the eye and adnexa	−0.55 (1.34; 0.24)	0.172		
Diseases of the circulatory system	−0.21 (− 0.56; 0.13)	0.220		
Diseases of the respiratory system	−0.04 (− 0.83; 00.75)	0.917		
Diseases of the digestive system	0.19 (−0.43; 0.83)	0.543		
Diseases of the musculoskeletal system and connective tissue	−2.42 (−4.99; 0.15)	0.066		
Interventions	−0.29 (−0.92; 0.32)	0.348		
DAMNP^4^				
Before deployment	1.00		1.00	
After deployment	−0.36 (−0.51; − 0.20)	< 0.001	− 0.20 (− 0.37; − 0.10)	< 0.001
CATS^5^				
Before deployment	1.00		1.00	
After deployment	−0.48 (− 066; − 0.31)	< 0.001	−0.25 (− 0.30; − 0.10)	< 0.001
			*R*^*2*^: 0.140	
			*R*^*2*^ adjusted: 0.132	

^1^Regression coefficient; ^2^Confidence interval of 95%;^3^Model adjusted by sex, age, origin of the prescription, quantity of medicines, neoplastic diseases, blood diseases, endocrine diseases, diseases of the osteomuscular system and strategies (DAMNP e CATS); ^4^Period before the deployment of ^5^Department of Assessment of Non-Standardized Medicines: 2003–2005/Period after the deployment of Department of Assessment of Non-Standardized Medicines: 2007–2015; ^5^Period before deployment of Technical Chamber of Health Assessment: 2003–2008/ Period after deployment of Technical Chamber of Health Assessment: 2010–2015

Table 8 shows a 19% reduction in the prevalence of medicines in the SUS formulary (APR:0.81; *p* = 0.032) and an increase in the prevalence of medicines outside the SUS formulary with a therapeutic alternative (APR:1.38; *p* = 0.011) after the deployment of the CATS. The effect of the two interventions was not verified by the proportion of lawsuits for medicines outside the SUS formulary and without a therapeutic alternative.

**Table 8:** Analysis of Poisson’s regression of the effects of the deployment of DAMNP and CATS on the prevalence of medicines that are within the SUS formulary, outside the SUS formulary with a therapeutic alternative and without therapeutic alternative available by the SUS

Variables	Medicines within the SUS formulary^c^		Medicines outside the SUS formulary with a therapeutic alternative^c^		Medicines outside the SUS formulary without a therapeutic alternative^c^	
	APR^a^ (95.0% CI)^b^	*p*	APR^a^ (95.0% CI)^b^	*p*	APR^a^ (95.0% CI)^b^	*p*
DAMNP^d^						
Before deployment	1.00		1.00		1.00	
After deployment	1.07 (0.93–1.24)	0.307	1.38 (0.80–1.29)	0.867	0.83 (0.63–1.34)	0.183
CATS^e^						
Before deployment	1.00		1.00		1.00	
After deployment	0.81 (0.67–0.98)	0.032	1.38 (1.07–1.78)	0.011	0.97 (0.70–1.34)	0.866

^a^Adjusted Prevalence ratio; ^b^Confidence interval of 95%; ^c^Model adjusted according to the origin of the prescription, DAMNP and CATS. ^d^Department of Assessment of Non-Standardized Medicines; ^e^Technical Chamber of Health Assessment

## Discussion

It was observed that institutional strategies that work in an interinstitutional manner are effective in mitigating some of the side effects of the judicialization of the right to health, such as a reduction in the filing of new lawsuits and that people who really need access to medicines now have more barriers. A similar reduction was also observed in a previous study in Brazil [30].

For the justice system, the reduction of the procedural burden involving requests for medicines can contribute to a reduction in the administrative cost of lawsuits, such as employee expenses, consumables, paper, etc. Although these costs are hard to measure, the Institute of Applied Economic Research (Instituto de Pesquisa Econômica Aplicada, or IPEA) determined that the cost of a first-degree tax enforcement lawsuit is US$2143.56, and this serves as a parameter for the cost of a lawsuit in the health area [47]. Therefore, the reduction of these costs allows for the allocation of the resources of the judiciary to cases that are not related to health care.

However, the reduction in the costs of the medicines in the lawsuits does not necessarily imply a reduction in spending by the secretaries of health for medicines outside of the paths institutionalized by public policy. In this case, although the executive branch is able to reduce the purchase of medicines by lawsuits, expenses still remain. What happens is a management and better control of what is being requested. The CATS can manage better because it can modify the prescription order and replace it with medicines in the SUS formulary, while in court this does not happen anymore: the judge orders the supply of the medicine, without the option to check if there is an available and cheaper alternative for the executive.

In addition, if the state remains inefficient in promoting the access to such items, the needs of the citizens will still remain, and these may motivate a shift in requests for medicines from lawsuits to administrative cases.

It is evident that a reduction in the costs of medicines from lawsuits benefits society, which is responsible for the financing of the health and justice systems in Brazil through social contributions and the payment of taxes. In this way, the deployment of institutional strategies favors the collective well-being and guarantees access to medicines in the SUS formulary and at a more acceptable cost to society.

The observed reduction in request for the medicines in the SUS formulary is related to the creation and deployment of institutional strategies by the municipality; the needs of the users were evaluated by a technical team that was knowledgeable about public health policies, had the ability to comprehend and assess the individual needs of the plaintiffs, and could provide feedback to instruct users about the SUS formulary for the requested medication, making litigation unnecessary. The two strategies prioritized the requests of citizens through institutionalized or administrative cases; therefore, the requests for medicines that were not included on the official lists were addressed using the legal route, as demonstrated by other studies [2, 3, 48].

However, the deployment of the CATS strategy increased requests for medicines outside the SUS formulary but with a therapeutic alternative. It was assumed that these institutional strategies would lower the number of these requests, because in their feedback, they proposed options for treatment with medications that had the same efficacy and were available through the SUS. This finding is likely a result of prescribing medicines in an uncritical manner, often without scientific evidence, or because of pressure from the pharmaceutical industry, or due to the prescribers’ nonadherence to the SUS formulary [49]. These situations indicate how pharmacological and economic irrationalities could be avoided if magistrates took advantage of the technical opinions of the CATS before filing a lawsuit, as recommended by the National Council of Justice [28].

The prevalence of requests for medicines outside the SUS formulary without a therapeutic alternative was not influenced by institutional strategies, proving the need to update policies and incorporate new technologies by the SUS. Legal demands for medicines are justified when the provision in the policies is not guaranteed or when the request involves medicines not covered by the policies and without a therapeutic alternative, reflecting gaps in care [4].

It is known that the process of incorporating new technologies is a determining factor in the increase in spending on health systems in many countries [50]. Accordingly, Brazil has passed law number 12,401/2011 [51] and decree number 7508/2011 [52], which introduced modifications and additions to law number 8080 of 1990 [53], referring to therapeutic assistance and the incorporation of technologies in the SUS. However, political and market pressures are still observed in favor of incorporating into the SUS formulary the medicines that are most requested in the lawsuits [54].

In relation to another political aspect of health judicialization, the increase in cases about prescriptions by an SUS clinician after the deployment of the CATS observed in this study demonstrated two possible situations. The first indicates that an improvement in access to medical appointments in the public system by the plaintiffs is responsible; the second entails the use of the SUS by users of the private system only to obtain a transcription of the prescription by a professional accredited to the SUS. This last situation has been a recurrent practice, in which users seek to overcome the lack of coverage of medications in the private system and exhibit a private-public mix, that is, they seek medical assistance that is guaranteed by the private system and access to medications provided by the public system [55].

With regard to the social aspects, it was observed that the profile of the plaintiffs filing the lawsuits did not change after the deployment of the strategies; the population was still characterized by a higher income, and they were privileged by their ability to afford doctor appointments in the private system and the expenses related to the lawsuits. Other studies have also suggested that health judicialization benefits those citizens with better economic status, and therefore it worsens the inequities in regard to access to health care [19, 56].

It is well known that health judicialization is a multidimensional problem, but the larger concern is the actual effectiveness of the pharmaceutical care provided by the health system. The management of the pharmaceutical service must reorient its actions and guarantee access to essential medications that are established in pharmaceutical policy according to the principles of the SUS. The justice system should only be used when this access is not guaranteed.

As a matter of fact, health judicialization brought changes in social and institutional relationships so as to guarantee the integrity of health care in the face of the social demands and deficiencies of the public health system. In addition, it created formal spaces for dialogue between the justice and health systems, which is fundamental for ensuring effective public policy, such as the DAMNP and CATS strategies. These strategies did not eliminate lawsuits, but they rationalized some economic and political distortions and normalized access to medicines outside the SUS formulary and took into consideration alternatives made available by the SUS and other clinical protocols.

The benefits generated by the deployment of institutional strategies should not be analyzed only from the perspective of the health system. The clinical rationality and the right to health benefits, physical integrity, and well-being aspects of the human dignity of the population should be considered. The Brazilian citizens who pay taxes and finances the public health system should expect that resources dedicated to health treatment be used in an adequate manner that benefits their own health and that of their families. Thus, it is important to assess at what point the economic interests of the state to reduce spending on health judicialization overcomes the real needs of the user.

With the implementation of institutional strategies, the needs of the users were evaluated by a technical team that was knowledgeable about public health policies and had the ability to comprehend and assess the individual needs of the citizens, proving the need to update policies and incorporate new technologies, with evidence, efficacy, and safety and which have lower costs. These actions improve access to medicines.

Hopefully, the results of this study can serve as a model for many health and justice systems in other countries where health judicialization is practiced, and these findings can contribute to restructured and enhanced policies, guaranteed access to essential medicines in an equitable manner and special attention to the needs of the people who depend on health systems for their therapeutic treatment.

## Data Availability

The datasets generated and/or analysed during the current study are not publicly available due to the data belong to the Pharmacy of Health-related Products and Special Medications that is a public institution where data of granted cases are kept. According to the regulations of the entity, data cannot be disseminated or shared but are available from the corresponding author on reasonable request.

## Abbreviations

APR: Adjusted Prevalence Ratio
ATC: Anatomical Therapeutic Chemical
CI: Confidence Interval
DAMNP: Department of Assessment of Non-Standardized Medicines
ICD: International Statistical Classification of Diseases and Related Health Problems; CATS Technical Chamber of Health Assessment
IQR: Interquartile Range
PR: Prevalence Ratio
SD: Standard Deviation
SUS: Brazilian Universal Health System
UTP: Territorial Planning Units

## References

[1] Instituto de Pesquisa Econômica Aplicada. Políticas Sociais: acompanhamento e análise, n. 14, 2007. http://www.ipea.gov.br/portal/images/stories/PDFs/politicas_sociais/bps14_completo.pdf. Accessed 21 Jan 2017.

[2] Reveiz L, Chapman E, Torres R, Fitzgerald JF, Mendoza A, Bolis M, Salgado O (2013). Right-to-health litigation in three Latin American countries: a systematic literature review. Revista Panamericana de Salud Publica.

[3] Vargas-Peláez CM, Rover MR, Leite SN, Rossi Buenaventura F, Farias MR (2014). Right to health, essential medicines, and lawsuits for access to medicines - A scoping study. Soc Sci Med.

[4] Pandolfo M, Delduque MC, Amaral RG (2012). Aspectos jurídicos e sanitários condicionantes para o uso da via judicial no acesso aos medicamentos no Brasil. Rev Salud Pública.

[5] Wang DWL, Vasconcelos NP, Oliveira VE, Terrazas FV (2014). Os impactos da judicialização da saúde no município de São Paulo: gasto público e organização federativa. Rev Adm Pública.

[6] Brasil. Constituição da República Federativa do Brasil de 1988. Diário Oficial da União, Brasília, 5 out. 1988. Seção I.

[7] Brasil. Portaria n° 3.916, de 30 de outubro de 1998. Dispõe sobre a aprovação da Política Nacional de Medicamentos. http//www.saude.gov.br/doc/portariagm3916/gm.htm. Accessed 20 Jan 2016.

[8] Brasil*.* Ministério da Saúde. Secretaria de Ciência, Tecnologia e Insumos Estratégicos. Departamento de Assistência Farmacêutica e Insumos Estratégicos. Relação Nacional de Medicamentos Essenciais: Rename 2017. Brasília: Ministério da Saúde; 2017. 210 p.

[9] Bertoldi AD, Helfer AP, Camargo AL, Tavares NUL, Kanavos P (2012). Is the Brazilian pharmaceutical policy ensuring population access to essential medicines?. Glob Health.

[10] Diniz D, Medeiros M, Schwartz IVD (2012). Consequências da judicialização das políticas de saúde: custos de medicamentos para as mucopolissacaridoses. Cad Saúde Pública.

[11] Barroso LR. Judicialização, Ativismo Judicial e Legitimidade Democrática]. (Syn) Thesis 2012; 5: 23–32.

[12] Boing AC, Bertoldi AD, Boing AF, Bastos JL, Peres KG (2013). Acesso a medicamentos no setor público: análise de usuários do Sistema Único de Saúde no Brasil. Cad Saúde Pública.

[13] Abadia CE, Oviedo DG (2009). Bureaucratic itineraries in Colombia: a theoretical and methodological tool to assess managed-care health care system. Soc Sci Med.

[14] Norheim F, Wilson BM (2014). Health rights litigation and access to medicines: priority classification of successful cases from Costa Rica’s constitutional chamber of the supreme court. Health Hum Rights.

[15] Zuniga FA (2014). When constitutional justice has the last word on health care: the case of Chile. Int J Health Serv.

[16] David G, Andrelino A, Beghin N (2016). Direito a medicamentos: Avaliação das despesas com medicamentos no âmbito federal do Sistema Único de Saúde entre 2008 e 2015.

[17] Conselho Nacional de Justiça. Justiça em números 2010. http://cnj.jus.br/images/programas/justica-em-numeros/rel_sintetico_jn2009.pdf/ Accessed 21 Jan 2017.

[18] Conselho Nacional de Justiça. Justiça em números 2011. http://cnj.jus.br/images/programas/justica-em numeros/2010/rel_justica_numeros_2010.pdf/ Accessed 21 Jan 2017.

[19] Machado MAA, Acurcio FA, Brandão CMR, Faleiro DR, Guerra AA, Cherchiglia ML, Andrade ALG (2011). Judicialização de acesso a medicamentos no Estado de Minas Gerais Brasil. Rev de Saúde Pública.

[20] Vieira FS, Zucchi P (2007). Distorções causadas pelas ações judiciais à política de medicamentos no Brasil. Rev de Saúde Pública.

[21] Pinzón-Flórez CE, Chapman E, Cubillos L, Reveiz L (2016). Prioritization of strategies to approach the judicialization of health in Latin America and the Caribbean. Rev Saúde Pública.

[22] Santos L, Terrazas F, Assis G (2014). Judicialização da saúde no Brasil. Mediação sanitária: direito, saúde e cidadania.

[23] Asensi F, Pinheiro R (2016). Judicialização da saúde e diálogo institucional: a experiência de Lages (SC). Rev de Direito Sanitário.

[24] Goiânia. Decreto n° 4051, de 02 de setembro de 2013. Aprova o Regimento Interno da Secretaria Municipal de Saúde, e dá outras providências*.* http://www.goiania.go.gov.br/Download/legislacao/diariooficial/2013/do_20130905_000005670.pdf. Accessed 15 July 2015.

[25] Ministério Público do Estado de Goiás. Ato PGJ n° 01/2014. Institui no âmbito do Ministério Público do Estado de Goiás, a Câmara de Avaliação Técnica em Saúde (CATS), órgão auxiliar do Centro de Apoio Operacional para emissão de parecer técnico acerca do fornecimento de medicamentos. http://www.mpgo.mp.br/portal/arquivos/2015/02/25/10_01_12_26_Ato_PGJ_n._01_2014_institui_C%C3%A2mara_de_Avalia%C3%A7%C3%A3o_T%C3%A9cnica_em_Sa%C3%BAde_CATS.pdf. Accessed 20 May 2015.

[26] Ministério Público do Estado de Goiás. Termo de Cooperação n° 044/2010 MPGO. Termo de Cooperação Técnica que entre si celebram o Ministério Público do Estado de Goiás e a Secretaria Municipal de Saúde de Goiânia, visando regular o procedimento para a dispensação de medicamentos, insumos e correlatos dos pacientes que obtiverem parecer favorável da Câmara de Avaliação Técnica de Saúde*.* http://www.mpgo.mp.br/portal/arquivos/2014/12/15/14_38_41_58_TAC_CATS.pdf/. Accessed 20 May 2015.

[27] Conselho Nacional de Justiça. Institui o Fórum Nacional do Judiciário para monitoramento e resolução das demandas de assistência à saúde. Resolução n° 107 de 6 de abril de 2010. http://www.cnj.jus.br/busca-atos-adm?documento=283. Accessed 08 May 2016.

[28] Conselho Nacional de Justiça. Recomendação n° 31 de 30 de março de 2010. http://www.cnj.jus.br/atos-administrativos/atos-da-presidencia/322-recomendacoes-do-conselho/12113-recomendacao-no-31-de-30-de-marco-de-2010. Accessed 21 Jan 2017.

[29] Asensi FD (2010). Judicialização ou juridicização? As instituições jurídicas e suas estratégias na saúde. Physis.

[30] Provin MP, Leite SN, Amaral RG (2013). Social inequalities in lawsuits for drugs. Braz J Pharm Sci.

[31] Instituto Brasileiro de Geografia e Estatística. Censo demográfico 2010*.* http://www.cidades.ibge.gov.br/xtras/temas.php?lang=&codmun=520870&idtema=108&search=goias|goiania|censo-demografico-2010:-resultados-da-amostra-rendimento%2D%2D. Accessed 16 May 2016.

[32] Centers for Disease Control and Prevention. International Classification of Diseases, Tenth Revision (ICD-10). www. cdc.gov/nchs/icd/icd10.htm. Accessed 16 May 2015.

[33] WHO Collaboration Center for Drug Statistics Methodology. Anatomical Therapeutic Chemical Classification. http://www.whocc.no/atc_ddd_index. Accessed 17 May 2015.

[34] Ministério da Saúde (2016). Banco de Preços em Saúde.

[35] Warriner AH (2014). Effect of self-referral on bone mineral density testing and osteoporosis treatment. Med Care.

[36] Arija V (2017). Effectiveness of a physical activity program on cardiovascular disease risk in adult primary health-care users: the “Pas-a-Pas” community intervention trial. BMC Public Health.

[37] Razali NM, Wah YB (2011). Power comparisons of Shapiro-Wilk, Kolmogorov-Smirnov, Lilliefors and Anderson-Darling tests. J Stat Model Anal.

[38] Diringer MN, Edwards DF, Mattson DT, Akins PT, Sheedy CW, Hsu CY (1999). Predictors of acute hospital costs for treatment of ischemic stroke in an academic center. Stroke..

[39] Schneider A, Hommel G, Blettner M (2010). Linear regression analysis: part 14 of a series on evaluation of scientific publications. Dtsch Arztebl Int.

[40] McGee CE, Trigwell J, Fairclough SJ, Murphy RC, Porcellato L, Ussher M (2016). Effect of a sport-for-health intervention (SmokeFree sports) on smoking-related intentions and cognitions among 9-10 year old primary school children: a controlled trial. BMC Public Health.

[41] Desgagné A, Castilloux AM, Angers JF, LeLorier J (1998). The use of the bootstrap statistical method for the pharmacoeconomic cost analysis of skewed data. Pharmacoeconomics..

[42] Efron B, Tibshirani T (1993). Introduction to the botstrap.

[43] Robinson C, Schumacker RE (2009). Interaction effects: centering, variance inflation factor, and interpretation issues. Mult Linear Regression Viewpoint.

[44] White H (1980). A heteroskedasticity- consistent covariancematix estimator and a direct test for heteroskedasticity. Econometrica.

[45] Coutinho LMS, Scazufca M, Menezes PR (2008). Methods for estimating prevalence ratios in cross-sectional studies. Rev Saúde Pública.

[46] Barreto ML, Genser B, Strina A, Teixeira MG, Assis AMO, Rego RF, Teles CA, Prado MS, Matos S, Alcântara-Neves NM, Cairncross S (2010). Impact of a citywide sanitation program in Northeast Brazil on intestinal parasites infection in young children. Environ Health Perspect.

[47] Instituto de Pesquisa Econômica Aplicada. Mapa da Defensoria 2013. http://www.ipea.gov.br/sites/mapadefensoria/desafios/Accessed 27 May 2016.

[48] Leite SN, Pereira SM, Silva P, Nascimento Júnior JM, Cordeiro BC, Veber AP (2009). Ações judiciais e demandas administrativas na garantia do direito de acesso a medicamentos em Florianópolis-SC. Revista de Direito Sanitário.

[49] Pepe VLE, Figueiredo TA, Simas L, Osorio-de-Castro CG, Ventura M (2010). Health litigation and new challeges in the management of pharmaceutical services. Ciência Saúde Coletiva.

[50] Nandakumar AK, Farag ME (2008). International encyclopedia of public health. Determinants National Health Expenditure.

[51] Brasil. Lei n.°12.401. Altera a Lei no 8.080 para dispor sobre a assistência terapêutica e a incorporação de tecnologia em saúde no âmbito do Sistema Único de Saúde-SUS. Diário Oficial da União, 2011; 29 April.

[52] Brasil*.* Decreto n° 7.508. Regulamenta a Lei n° 8.080, de 19 de setembro de 1990, para dispor sobre a organização do Sistema único de Saúde – SUS, o planejamento da saúde, a assistência à saúde e a articulação interfederativa, e dá outras providências. Diário Oficial da União, 2011; 29 June.

[53] Brasil. Lei n.°8.080. Dispõe sobre as condições para promoção, proteção e recuperação da saúde, a organização e o funcionamento dos serviços correspondentes e dá outras providências. Diário Oficial da União, 1990; 20 Sept.

[54] Santos-Pinto CDB, Ventura M, Pepe VLE, Osorio-de-Castro CGS (2013). Novos delineamentos da Assistência Farmacêutica frente à regulamentação da Lei Orgânica da Saúde. Cad Saúde Pública.

[55] Leite SN, Mafra AC (2010). Que direito? Trajetórias e percepções dos usuários no processo de acesso a medicamentos por mandados judiciais em Santa Catarina. Ciênc Saúde Coletiva.

[56] Chieffi AL, Barata RB (2009). Judicialização da política pública de assistência farmacêutica e equidade. Cad Saúde Pública..

